# Auxin signaling: a big question to be addressed by small molecules

**DOI:** 10.1093/jxb/erx375

**Published:** 2017-11-18

**Authors:** Qian Ma, Peter Grones, Stéphanie Robert

**Affiliations:** Umeå Plant Science Centre, Department of Forest Genetics and Plant Physiology, Swedish University of Agricultural Sciences, Sweden

**Keywords:** Auxin, auxin response, auxin signaling, chemical biology, chemical genetics, phytohormones, small molecules

## Abstract

Providing a mechanistic understanding of the crucial roles of the phytohormone auxin has been an important and coherent aspect of plant biology research. Since its discovery more than a century ago, prominent advances have been made in the understanding of auxin action, ranging from metabolism and transport to cellular and transcriptional responses. However, there is a long road ahead before a thorough understanding of its complex effects is achieved, because a lot of key information is still missing. The availability of an increasing number of technically advanced scientific tools has boosted the basic discoveries in auxin biology. A plethora of bioactive small molecules, consisting of the synthetic auxin-like herbicides and the more specific auxin-related compounds, developed as a result of the exploration of chemical space by chemical biology, have made the tool box for auxin research more comprehensive. This review mainly focuses on the compounds targeting the auxin co-receptor complex, demonstrates the various ways to use them, and shows clear examples of important basic knowledge obtained by their usage. Application of these precise chemical tools, together with an increasing amount of structural information for the major components in auxin action, will certainly aid in strengthening our insights into the complexity and diversity of auxin response.

## Teaser

This review summarizes the current knowledge of auxin signaling and emphasizes how chemical genomics could help to unravel the complexity of the TIR1/AFB–Aux/IAA co-receptor system.

## Introduction

**Fig. 1.**
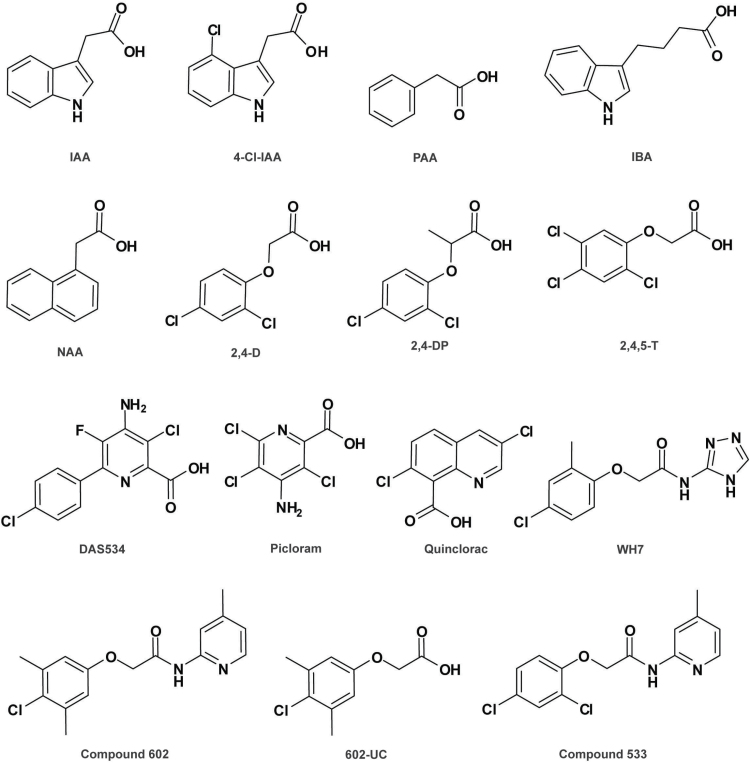
Structures of naturally occurring auxins and synthetic auxin agonists. The chemical names for the displayed compounds are: indole-3-acetic acid (IAA), 4-chloroindole-3-acetic acid (4-Cl-IAA), phenylacetic acid (PAA), indole-3-butyric acid (IBA), naphthalene-1-acetic acid (NAA), 2,4-dichlorophenoxyacetic acid (2,4-D), 2-(2,4-dichlorophenoxy) propionic acid (2,4-DP), 2,4,5-trichlorophenoxyacetic acid (2,4,5-T), 4-amino-3-chloro-6-(4-chlorophenyl)-5-fluoro-pyridine-2-carboxylic acid (DAS534), 4-amino-3,5,6-trichloropicolinic acid (picloram), 3,7-dichloro-8-quinolinecarboxylic acid (quinclorac), 2-(4-chloro-2-methylphenoxy)-*N*-(4-*H*-1,2,4-triazol-3-yl) acetamide (WH7), 2-(4-chloro-3,5-dimethylphenoxy)-*N*-(4-methylpyridin-2-yl) acetamide (compound 602), 2-(4-chloro-3,5-dimethylphenoxy) acetic acid (602-UC), and 2-(2,4-dichlorophenoxy)-*N*-(4-methylpyridin-2-yl) acetamide (compound 533).

Auxin is such a crucial phytohormone that it regulates almost every aspect of plant growth and development (Enders and Strader, 2015; Paque and Weijers, 2016). The conceptual hypothesis and discovery of auxin can be traced back for more than 140 years and directly originate from investigations of plant tropic growth responses. Among others, Charles Darwin, Arpád Paál, and Frits Went studied the phototropic response of coleoptiles of etiolated canary grass or *Avena* seedlings and hypothesized that a mobile signal from the tip induced by unilateral illumination is transmitted asymmetrically to the lower region beneath the tip, where it prompts curvature toward the light source due to differential elongation of one side of the coleoptiles (Darwin, 1880; Paál, 1914; Went, 1926). The term auxin, originated from the Greek word ‘auxein’ meaning ‘to grow or to expand’, was coined to describe substances with signaling activity based on the coleoptile curvature bioassay. During the 1930s, three auxin mimics, called auxin *a*, auxin *b* (Kögl and Haagen-Smit, 1931), and heteroauxin (Kögl *et al.*, 1934; Kögl and Kostermans, 1934; Thimann, 1935), were isolated from human urine and fungal cultures. The subsequent analyses finally identified heteroauxin, chemically determined to be indole-3-acetic acid (IAA) (Fig. 1), as the actual auxin. In the early 1940s, IAA was discovered for the first time in a higher plant, maize (Haagen-Smit *et al.*, 1942), and since then it has been widely recognized as the principal auxin in all plant species (Abel and Theologis, 2010).

By a strict definition, auxin is IAA, a simple molecule consisting of an indole ring and a carboxylic acid side chain, structurally similar to the amino acid tryptophan. However, in a broader sense, auxin comprises those compounds that can elicit a response analogous to what IAA does (Paque and Weijers, 2016). So far three other naturally occurring endogenous auxins have been discovered in plants, namely indole-3-butyric acid (IBA), 4-chloroindole-3-acetic acid (4-Cl-IAA) and phenylacetic acid (PAA) (Fig. 1), which are active in bioassays (Simon and Petrášek, 2011). Soon after the structural characterization of IAA, a diverse group of synthetic compounds, such as naphthalene-1-acetic acid (NAA), 2,4-dichlorophenoxyacetic acid (2,4-D), and picloram (Fig. 1), were identified as auxin-like substances and have since been widely used as chemical tools in studies of auxin biology and as plant growth regulators in agriculture and horticulture.

Auxin effects in the regulation of plant growth and development are largely determined by the coordination of three complex processes: auxin metabolism, auxin transport, and auxin signaling. In Arabidopsis, IAA is mainly synthesized from tryptophan via the indole-3-pyruvic acid pathway (Mashiguchi *et al.*, 2011) and deactivated primarily through conjugation to small molecules (Ludwig-Müller, 2011; Korasick *et al.*, 2013) and by oxidation to 2-oxindole-3-acetic acid (Mellor *et al.*, 2016; Porco *et al.*, 2016; Zhang *et al.*, 2016). Auxin transport in plants is facilitated by three protein families: the PIN-FORMED and ATP-BINDING CASSETTE SUBFAMILY B families facilitate IAA efflux, whereas the AUXIN RESISTANT1/LIKE AUX1 transporters facilitate IAA uptake (Zazimalova *et al.*, 2010, Peer *et al.*, 2011). Localized auxin metabolism and directional intercellular auxin transport together cause the formation of a differential auxin distribution or an auxin gradient in a given tissue, which is believed to determine individual cell responses to the hormone (Vanneste and Friml, 2009). The cell interprets auxin gradient information through a short nuclear signaling pathway, triggering cell type-specific transcriptional or cellular responses that consequently modulate plant growth and development (Fendrych *et al.*, 2016; Weijers and Wagner, 2016).

The core components of this auxin signaling pathway, which have been well characterized, are the F-box-containing TRANSPORT INHIBITOR RESISTANT1/AUXIN SIGNALING F-BOX (TIR1/AFB) proteins, the transcriptional co-repressors called AUXIN/INDOLE-3-ACETIC ACID (Aux/IAA), and the transcription factors AUXIN RESPONSE FACTOR (ARF). Auxin binds with the receptor TIR1/AFB, which is the substrate-recognition subunit of the SUPPRESSOR OF KINETOCHORE PROTEIN 1 (SKP1)/CULLIN1/F-Box (SCF) E3 ubiquitin ligase complex, and promotes the interaction between TIR1/AFB and Aux/IAA, triggering polyubiquitination and degradation of the Aux/IAA. The removal of Aux/IAA allows ARF to activate or repress early auxin-responsive gene transcription (Weijers and Wagner, 2016). Auxin here behaves like a molecular glue that is perceived by the SCF^TIR1/AFB^–Aux/IAA co-receptor system to trigger transcriptional reprogramming (Tan *et al.*, 2007; Calderón-Villalobos *et al.*, 2012), which is the main driver for auxin responses.

An enigmatic but fascinating question in auxin biology is how a relatively simple molecule (IAA) could exert such diverse effects? One crucial contribution to diversification in auxin response may lie in the property of the compact nuclear auxin perception and response system described before. In plants, the three central components of this system are encoded by multigene families, with six TIR1/AFBs, 29 Aux/IAAs, and 23 ARFs in Arabidopsis (Dharmasiri *et al.*, 2005*b*; Okushima *et al.*, 2005; Overvoorde *et al.*, 2005; Parry *et al.*, 2009). On the one hand, differential expression patterns for the members of each component can define a development- and/or tissue-specific assembly of the nuclear auxin perception and response system. On the other hand, the intrinsic biochemical affinities for interactions among the three core components and between ARFs and the promoters of the cognate target genes are very different (Vernoux *et al.*, 2011; Calderón-Villalobos *et al.*, 2012; Boer *et al.*, 2014; Lee *et al.*, 2014; Piya *et al.*, 2014; Shimizu-Mitao and Kakimoto, 2014; Dinesh *et al.*, 2016; Winkler *et al.*, 2017), which may be a cornerstone that imparts dynamic range and specificity to the system (Paque and Weijers, 2016). Especially, formation of a stable ternary TIR1/AFB–auxin–Aux/IAA co-receptor complex is crucial for the following signal transducing reactions. Different TIR1/AFB–Aux/IAA combinations exhibit different affinities for one another and for different auxins, including both natural and synthetic ones (Calderón-Villalobos *et al.*, 2012; Lee *et al.*, 2014; Shimizu-Mitao and Kakimoto, 2014). These varying affinities and specificities generated by the combinatorial availability of the type and amount of auxin and the sets of TIR1/AFB–Aux/IAA present in a given tissue or cell may lead to the differential clearance of Aux/IAA proteins and consequently the differential activation of ARFs, enabling the auxin response machinery to have the potential to generate various responses according to contextual clues (Enders and Strader, 2015; Paque and Weijers, 2016; Winkler *et al.*, 2017).

To unravel the mechanisms of auxin perception and response, a plethora of synthetic compounds have been developed to mimic or perturb the behavior of endogenous auxin to interrogate its effects, serving as a complementary approach to the canonical genetic and genomic approaches (De Rybel *et al.*, 2009; Ma and Robert, 2014). This chemical biology approach is based on the idea of using bioactive small molecules to dissect and understand biological systems. Its rationale is further supported by the auxin co-receptor model and the recent finding from the high-resolution structural analysis of the auxin co-receptor complex that the auxin-binding pocket of TIR1 adopts a structure/shape specific for auxinic molecules while maintaining partial promiscuity, providing both selectivity and plasticity towards ligand binding. Diverse compounds can be developed to fit into the auxin-binding pocket of the TIR1/AFB–Aux/IAA co-receptor system analogously to IAA that will exert differential effects on the interactions between TIR1/AFB and Aux/IAA. This will lead to modulation of ubiquitination and degradation rates of Aux/IAA proteins, which are thought of as one of the crucial mechanisms to define the specific auxin responses (Havens *et al.*, 2012; Pierre-Jerome *et al.*, 2014). In this review, we will first summarize the current knowledge on naturally occurring auxins and the core auxin response module in plants. Then we will focus on the chemical tools targeting the auxin co-receptor system and how their applications in conjunction with modern genetic and genomic tools can help unravel the mechanisms of the diverse auxin responses.

## Natural auxins

### Several types of natural auxins in plants

Since its discovery, auxin has been found to be fundamental for the regulation of various developmental processes, such as leaf abscission (Rubinstein and Leopold, 1963; Hepworth and Pautot, 2015), fruit formation (De Jong *et al.*, 2009; Łangowski *et al.*, 2016), abiotic stress (Liu *et al.*, 2014*b*), response to pathogens (Fu and Wang, 2011; Kazan and Lyons, 2014; Spaepen and Vanderleyden, 2011), senescence (Kim *et al.*, 2016), establishment and maintenance of polarity or apical dominance (Shao and Dong, 2016; Van Norman, 2016; Pillitteri *et al.*, 2016) and establishment and maintenance of tropic responses toward light and gravity (Armengot *et al.*, 2016; Carriedo *et al.*, 2016; Ballaré and Pierik, 2017). Moreover, the auxin signaling pathway also interacts with the signaling pathways of other phytohormones and therefore influences almost all plant development processes, directly or via cross-talk (Liu *et al.*, 2014*a*; Chandler and Werr, 2015; Hu *et al.*, 2017).

Endogenous auxin or IAA is responsible for the regulation of proper plant growth and development (Zhao, 2010); however, besides IAA, three additional compounds with a similar structure are naturally present in plants. IBA, a naturally occurring auxin precursor, was first identified in potato (*Solanum tuberosum*) tubers (Blommaert, 1954), and since then has been detected in many other plant species (Korasick *et al.*, 2013). Despite the IBA levels in Arabidopsis being below the current detection limit (Novák *et al.*, 2012), there are certain reasons to think it might play a role in plant development. IBA can be a source of IAA and vice versa, meaning that IBA might act as a biologically inactive storage form of IAA (Bartel *et al.*, 2001; Woodward and Bartel, 2005). Conversion of IBA to IAA is facilitated by peroxisomal β-oxidation enzymes such as INDOLE-3-BUTYRIC ACID RESPONSE1 (IBR1; Zolman *et al.*, 2008), IBR3 (Zolman *et al.*, 2007), IBR10 (Zolman *et al.*, 2008), and ENOYL-COA HYDRATASE2 (Strader *et al.*, 2011). Mutants in these enzymes exhibit decreased IAA levels, and consequently a shorter root meristem, less lateral roots, a reduced apical hook curvature and smaller cotyledons (Zolman *et al.*, 2008; Strader *et al.*, 2010; Strader *et al.*, 2011). The transport of IBA is facilitated by the ATP-BINDING CASSETTE SUBFAMILY G (ABCG) protein family of efflux carriers. To date, ABCG36 and ABCG37 are the only family members that have been characterized as being able to transport IBA, but not IAA (Ito and Gray, 2006; Strader *et al.*, 2008; Strader and Bartel, 2009; Růžička *et al.*, 2010). Finally, IBA to IAA conversion in the root cap is necessary for creating a local auxin source crucial for the oscillating transcriptional mechanism responsible for the regular spacing of lateral roots (De Rybel *et al.*, 2012; Xuan *et al.*, 2015).

First discovered in pea (*Pisum sativum*) seeds (Gandar and Nitsch, 1967; Marumo *et al.*, 1968), 4-Cl-IAA has been subsequently detected in many other plant species, mostly in members of the family Fabaceae (Engvild, 1975, 1980; Engvild *et al.*, 1978; Hofinger and Böttger, 1979; Katayama *et al.*, 1987). As its stability is higher than IAA, 4-Cl-IAA is active at lower concentrations (Marumo and Yamamoto, 1973). Despite the presence of 4-Cl-IAA having not yet been proven in Arabidopsis, various bioassays confirm its auxinic activity after exogenous application; for example 4-Cl-IAA can stimulate auxin-responsive promoter elements and cannot inhibit growth of *tir1* mutant (Simon *et al.*, 2009). Moreover, in pea 4-Cl-IAA stimulates pericarp growth (Reinecke *et al.*, 1995) and in maize it is responsible for coleoptile elongation, protoplast swelling and maintenance of membrane potential (Rescher *et al.*, 1996; Steffens and Lüthen, 2000; Karcz and Burdach, 2002).

The third naturally occurring auxin-like compound is PAA. It is at present the only identified IAA phenyl derivative. As observed *in vitro*, active concentrations of PAA are much higher than those of IAA (Fitzsimons, 1989; Small and Morris, 1990). PAA has been found in many different plant species at various concentration ranges (Wightman and Lighty, 1982) and it has been shown that PAA can interfere with active efflux of IAA in pea, thus playing a role in plant root interaction with soil microorganisms (Morris and Johnson, 1987; Slininger *et al.*, 2004; Somers *et al.*, 2005).

### Role of conjugation and metabolism in auxin homeostasis

Due to the importance of auxin throughout overall plant development, precise regulation of its levels during plant growth is required. This can be achieved by either symplastic transport of auxin from a place of high concentration (using cellular auxin importers and exporters) or local inactivation of its function (via degradation or conjugation).

IAA’s local and reversible inactivation by coupling with amino acids, sugars or proteins has been considered as an efficient way to rapidly regulate hormone contents. Such coupled IAA molecules can be stored or transported over long distances (Wood, 1985). Two major conjugation mechanisms of IAA have been identified: formation of amide bonds with amino acids, proteins, and peptides, and formation of ester bonds with glucan, inositol, indole acetyl glucose and glycoproteins. Amide bond formation is organized by the multiprotein family GRETCHEN HAGEN 3 (GH3; Hagen and Guilfoyle, 1985; Wright *et al.*, 1987; Li *et al.*, 1991). The overexpression of *GH3* genes in Arabidopsis causes a dwarf phenotype due to a reduced level of IAA and the loss-of-function mutants exhibit increased auxin sensitivity by stronger auxin-mediated root growth inhibition (Staswick *et al.*, 2005). The formation of ester bonds is catalysed by IAA CARBOXYL METHYLTRANSFERASE 1, the overexpression of which leads to a hyponastic leaf phenotype (Qin *et al.*, 2005).

On the other hand, an irreversible mechanism of auxin inactivation is its degradation by peroxidases. Two oxidation metabolites, 2-oxindole-3-acetic acid and 2-oxindole-3-acetic acid glucose, have been shown to increase in the wild-type after auxin treatment or in plants overproducing IAA (Östin *et al.*, 1998; Kai *et al.*, 2007; Stepanova *et al.*, 2011; Novák *et al.*, 2012). The recently characterized *DIOXYGENASE FOR AUXIN OXIDATION 1/2* (*DAO1/2*) plays a major role in auxin oxidation. *DAO1* and *DAO2* together with *GH3* orchestrate the homoeostasis of auxin, and interestingly, auxin oxidation is more pronounced at lower hormone concentrations, whereas auxin conjugation is most significant at high auxin levels (Mellor *et al.*, 2016; Porco *et al.*, 2016; Zhang *et al.*, 2016). Nevertheless, the whole molecular mechanisms and genes involved in IAA catabolism still remain to be unraveled.

### Role of auxin in response to environmental stresses

A key feature of plant growth and development, which is facilitated by plant hormones, is the adaptation to endogenous and environmental stimuli. Besides the well-recognized roles of abscisic acid, brassinosteroids, salicylic acid and gibberellins in these processes, auxin also plays an important role in plant adaptation.

Salt stress reduces plant growth and photosynthesis leading to accelerated aging and death (Golldack *et al.*, 2014). It has been shown that salt stress increases IAA levels in maize (Ribaut and Pilet, 1994), but reduces them in rice, tomato, and wheat (Prakash and Prathapasenan, 1990; Nilsen and Orcutt, 1997; Shakirova *et al.*, 2003), and thus the involvement of auxin in this response still remains to be properly evaluated. Salt-mediated root growth inhibition is caused by depletion of auxin from the root tip and inhibition of IAA17 degradation (Liu *et al.*, 2015; Jiang *et al.*, 2016). Auxin conjugation seems to also be involved in the salt stress response, as *GH3* gene expression levels in sorghum were increased in salt stress conditions (Wang *et al.*, 2010).

Until now, only a few studies have shown that besides abscisic acid (ABA), auxin plays a significant role in drought stress tolerance. In general, higher levels of endogenous auxin enhance drought stress resistance, by modulating root architecture, reactive oxygen species metabolism, and ABA-responsive gene expression (Lee and Luan, 2012; Kim *et al.*, 2013; Shi *et al.*, 2014; Cha *et al.*, 2015). IAA conjugates also participate in plant drought tolerance, as overexpression of *GH3* in rice or mutation of the *IAA ALANINE RESISTANT 3* (*IAR3*) gene increased plant sensitivity to drought (Du *et al.*, 2012; Kinoshita *et al.*, 2012).

Besides jasmonates, salicylic acid and ethylene, pathogen susceptibility is also controlled by auxin (Kazan & Lyons, 2014). Various pathogens target different parts of the auxin machinery, including auxin biosynthesis, signaling or transport. IAA has been shown to influence the expression of bacterial genes; for example in *Agrobacterium tumefaciens* IAA inhibits expression of the *vir* gene, which *A. tumefaciens* uses as a signaling mechanism for successful T-DNA transfer (Liu and Nester, 2006). Moreover, a plant defense response against *Pseudomonas syringae* attack includes stabilization of the Aux/IAA proteins and downregulation of the auxin-signaling pathway (Navarro *et al.*, 2006). In order to overcome this strategy, *P. syringae* produces the type III effector AvrRpt2, which interferes with the auxin signaling pathway by promoting the degradation of Aux/IAA proteins (Chen *et al.*, 2007; Cui *et al.*, 2013). Analogously, tobacco mosaic virus replicase proteins directly interfere with Aux/IAA proteins and alter their subcellular localization and stability (Padmanabhan *et al.*, 2005, 2008), thus increasing pathogen susceptibility.

## The core auxin response module

Following perception in the nucleus, auxin can trigger broad and specific transcriptional responses. The key components of the nuclear auxin signaling machinery are the TIR1/AFB F-box proteins, the Aux/IAA transcriptional repressors and the transcription factors ARF (Abel *et al.*, 1995, 1996; Ulmasov *et al.*, 1997, 1999; Remington *et al.*, 2004; Dharmasiri *et al.*, 2005*a*; Kepinski and Leyser, 2005; Calderón-Villalobos *et al.*, 2012).

The TIR1/AFB auxin receptor family comprises TIR1 and AFB1 to -5 and all of them have been show to function as auxin receptors (Dharmasiri *et al.*, 2005*a*,b; Kepinski and Leyser, 2005; Parry *et al.*, 2009). Single mutants in these genes display mild auxin-related phenotypes and only the *tir1* mutant exhibits auxin resistance and a shorter root (Ruegger *et al.*, 1998). On the other hand, higher order mutants especially combining *TIR1*, *AFB1*, *AFB2*, or *AFB3* show an increased auxin resistance and various growth defects (Dharmasiri *et al.*, 2005*b*; Parry *et al.*, 2009). *AFB4* and *AFB5* have diverged significantly from the other members of the TIR1/AFB family (Parry *et al.*, 2009). They have an extension on the N-terminal end of the protein and bind to the synthetic auxin picloram unlike the other members of the family (Calderón Villalobos *et al.*, 2012; Prigge *et al.*, 2016).

The basic structure of all the TIR1/AFB family members consists of a region of 18 leucine-rich-repeats (LRR) at the N-terminus and an F-box domain at the C-terminus. The crystal structure of TIR1 in complex with the SCF complex protein ARABIDOPSIS SKP1 HOMOLOGUE (ASK1), a small Aux/IAA peptide from IAA7, and auxin revealed that the auxin binding pocket is formed by the TIR1 LRR domain, while the TIR1 F-box domain interacts with ASK1 (Tan *et al.*, 2007). The single pocket for auxin binding is located at the top surface of the LRR region and facilitates both auxin perception and Aux/IAA recruitment. The structure of the binding site displays a quite promiscuous character, and thus it can accommodate different auxin-like compounds beside auxin itself (Tan *et al.*, 2007).

All the members of the *TIR1*/*AFB* family are specifically expressed in certain plant tissues throughout the life and their level of expression varies; for example in Arabidopsis *AFB1* is highly expressed in roots, *AFB3* in cotyledons or leaves, and *AFB5* in the hypocotyl (Dharmasiri *et al.*, 2005*b*; Parry *et al.*, 2009; Prigge *et al.*, 2016). Despite the fact that their domain of expression sometimes overlaps, the TIR1/AFBs are still able to facilitate specific auxin-mediated responses due to differential affinity to auxin and Aux/IAAs (Calderón-Villalobos *et al.*, 2012; Lee *et al.*, 2014; Shimizu-Mitao and Kakimoto, 2014). For example, the affinity (*K*_d_) of the TIR1–IAA7 pair for IAA is 10–15 nM, whereas the affinity of the TIR1–IAA12 pair for IAA is about 250–300 nM (Calderón-Villalobos *et al.*, 2012). A Recent study characterizing homologous IAA6 and IAA19 showed that despite a subtle sequence divergence between these two Aux/IAAs, IAA19 associates more strongly with TIR1/AFBs and is ubiquitylated with higher processivity at lower auxin concentrations compared with IAA6 (Winkler *et al.*, 2017). This suggests that a similar auxin concentration in different cells and/or different auxin concentrations in the same cell might lead to specific developmental responses. In this way, the co-receptor mechanism can expand the dynamic range of auxin perception and auxin-mediated response.

Another level of complexity is added on the level of post-transcriptional and post-translational modifications. MicroRNAs specific to TIR1/AFB family members have been identified, such as miR393, which negatively regulates *TIR1*, *AFB2*, and *AFB3* transcripts (Navarro *et al.*, 2006; Parry *et al.*, 2009; Vidal *et al.*, 2010; Si-Ammour *et al.*, 2011). It was shown that the expression of *miR393* can be controlled by various environmental stimuli such as the presence of salt or nitrate, establishing a link between environmental conditions and auxin regulation of plant development (Vidal *et al.*, 2010; Mendoza-Soto *et al.*, 2012; Iglesias *et al.*, 2014). TIR1 proteins can be post-translationally modified by nitric oxides through *S*-nitrosylation, which enhances TIR1–Aux/IAA interaction and promotes Aux/IAA degradation via the SCF^TIR1/AFB^ complex (Terrile *et al.*, 2012). It is suggested that this modification influences TIR1 oligomerization, which was shown to be crucial for effective accommodation and degradation of diverse Aux/IAA proteins (Dezfulian *et al.*, 2016). Additionally, the levels of TIR1 protein in the cell are closely regulated by autocatalytic degradation through the SCF complex (Stuttmann *et al.*, 2009; Yu *et al.*, 2015). All these above-mentioned regulatory mechanisms are essential for proper auxin-mediated plant development.

The Aux/IAA protein family significantly contributes to the diversity and complexity of auxin-mediated responses. Structurally, Aux/IAAs comprise several domains: domain I possessing an ETHYLENE RESPONSIVE ELEMENT BINDING FACTOR-ASSOCIATED REPRESSOR (EAR) motif mediating interaction with the TOPLESS co-repressor, domain II carrying a degron region that is responsible for interaction with TIR1/AFBs and a Phox and Bem 1 (PB1) domain essential for oligomerization with ARFs during transcriptional repression.

The primary amino acid sequences of the degron motif vary between different Aux/IAAs (some even lack the degron completely), and determine their overall stability (Dreher *et al.*, 2006). An important regulatory element is a conventional conserved pair of amino acids (lysine and arginine – KR) between domains I and II. Aux/IAAs possessing both the degron and KR motifs have an increased affinity towards the SCF complex, and therefore even low concentrations of auxin facilitate their degradation. In this way, the life time of Aux/IAA can vary from a few minutes (IAA5 or IAA7) to hours (IAA20 or IAA30) (Worley *et al.*, 2000; Gray *et al.*, 2001; Ouellet *et al.*, 2001; Ramos *et al.*, 2001; Zenser *et al.*, 2001; Dreher *et al.*, 2006;Calderón-Villalobos *et al.*, 2012; Havens *et al.*, 2012; Shimizu-Mitao and Kakimoto, 2014; Lee *et al.*, 2014; Moss *et al.*, 2015). Some Aux/IAAs, such as IAA5, IAA7, IAA9 or IAA17, which have a high sensitivity to auxin, tend to be degraded even in the absence of auxin (Shimizu-Mitao and Kakimoto, 2014). Mutations in the degron motif and its surroundings lead to Aux/IAA insensitivity to auxin and induce gain-of-function phenotypes (Rogg *et al.*, 2001; Fukaki *et al.*, 2002; Ploense *et al.*, 2009; Rinaldi *et al.*, 2012).

Similarly to TIR1/AFBs, Aux/IAAs can be also post-transcriptionally regulated. The identified microRNA miR847, which can be transcriptionally upregulated by auxin via TIR1, targets the mRNA of *IAA28* (Wang and Guo, 2015). In this way, auxin is not only responsible for degradation of IAA28 protein via the SCF^TIR1/AFB^ complex, but also its mRNA. Another type of modification involves Aux/IAA interaction with PHYTOCHROME A and phosphorylation by its protein kinase activity. It is suggested that such a modification might modulate Aux/IAA nuclear localization, metabolic stability and ability to interact with other Aux/IAA or ARF proteins (Colón-Carmona *et al.*, 2000). Yet another kind of Aux/IAA modification is isomerization of proline residues localized in domain II, which leads to decrease sensitivity of Aux/IAA proteins to the SCF^TIR1^ complex (Dharmasiri *et al.*, 2003). To date, the phosphorylation and isomerization of Aux/IAA proteins have only been demonstrated *in vitro*, and therefore the impact of these modifications on auxin responses still remains to be properly evaluated *in planta*. Furthermore, the oligomerization of Aux/IAAs was shown to play an important role in an efficient repression of ARF activity. The affinity of TOPLESS or histone deacetylases increases in the presence of Aux/IAA oligomer binding to ARF. This results in compaction of the chromatin environment, leading to DNA condensation and prevention of auxin-responsive gene expression (Korasick *et al.*, 2014; Han *et al.*, 2014).

As described above, the SCF^TIR/AFB^ auxin-responsive system is complex machinery, with redundancy in all of the basic components, TIR1/AFBs, Aux/IAAs and ARFs. Single loss-of-function mutants usually do not have any physiological phenotype, and therefore it is difficult to unveil the specific role of each of protein. The common strategy of creating high order mutants by genetic crossing is highly time-consuming and not always effective. Hence, chemical biology, with the ability to directly target single components by a specific chemical, can be a bright light at the end of the tunnel.

## Chemical probes targeting auxin perception machinery

Auxin research has always been intertwined with the use of various bioactive small molecules, including many synthetic auxin analogues, antagonists and biosynthesis and transport inhibitors. They are used to probe specific aspects of auxin action (De Rybel *et al.*, 2009; Ma and Robert, 2014; Doyle *et al.*, 2015). In the early stages of auxin research, a diverse group of auxin-like compounds, either synthetic or natural, were discovered from the structure–activity relationship studies based on bioassays (Zimmerman and Wilcoxon, 1935; Bentley, 1950; Hansch and Muir, 1950; Muir and Hansch, 1953). Some of those compounds have been and are still widely used today, although their mode of action has not been known until recently. The effects of most of these compounds are pleiotropic, which complicates the interpretation of results. With the advance of chemical biology in plant research, compounds with higher specificity targeting selective processes of auxin action have been developed through some elegant forward chemical genetic screens based on specific auxin-related readouts. Furthermore, structural insights into the TIR1/AFB–Aux/IAA–ARF nuclear auxin response module provide an unprecedented situation where reverse chemical genetic screens, established on the rational structure-based molecular design, are able to isolate potent small molecules targeting specific nodes of the auxin response.

### Synthetic auxins and auxin agonists

As part of the first generation of auxin-like compounds synthesized in the 1940s, NAA and 2,4-D are the most frequently used synthetic auxins in basic research and biotechnological applications. In the early years of auxin research when biochemical and physiological assays prevailed, they contributed to the structure–activity relationship studies aiming to reveal the defining characteristics of auxin molecules, which is still elusive and under investigation to this day. Later, based on the comprehensive analyses of diverse molecular structures of synthetic auxins, a common overall organization of auxin structure was postulated to consist of a planar aromatic ring system separated from a carboxyl group in a defined spatial position and distance (Ferro *et al.*, 2010). The key role of the two important functional moieties for auxin binding to the co-receptor has been elucidated by the crystal structure analysis of the TIR1–auxin–IAA7 DII degron complex (Tan *et al.*, 2007). A single auxin binding pocket of TIR1 is located on the top surface of the TIR1 LRR domain and is analogous to a three-walled room with an open ceiling. The planar ring of auxin stacks on top of the pocket floor with its edge packing against the surrounding walls through hydrophobic interactions. Meanwhile, the carboxyl group anchors the auxin molecule to the bottom of the pocket by forming a salt bridge and two hydrogen bonds with two polar residues on the pocket floor (Tan *et al.*, 2007). The Aux/IAA degron peptide binds to the auxin-bound TIR1 pocket through extensive hydrophobic interactions, completely covering the open ceiling. Auxin, acting as a molecular glue, enhances the binding affinity between TIR1 and Aux/IAA by filling a cavity between the two proteins, thereby extending the hydrophobic protein-interaction interface (Tan *et al.*, 2007).

The crystallographic analysis of the TIR1–IAA7 DII degron in complex with NAA and 2,4-D provided a unique chance to understand why a single auxin binding pocket can perceive the signals from diverse synthetic auxin-like molecules. Intriguingly, despite their distinct structures, NAA and 2,4-D bind to TIR1 in a similar manner to that of IAA, implying the auxin binding site in TIR1 is partially promiscuous, as mentioned previously. A more detailed comparison of their modes of binding reveals that an extra hydrogen bond formed between the unique NH group in the indole ring of IAA and a nearby carbonyl group of the TIR1 backbone, which is missing in NAA and 2,4-D, might be the cause of higher affinity of IAA to TIR1 in comparison with the other two compounds (Tan *et al.*, 2007). It is therefore postulated that the differences in the ring structures of auxin analogues might account for their different binding affinities to TIR1 (Calderón-Villalobos *et al.*, 2010). The partial promiscuity of the auxin-binding site offers the opportunity to modulate the auxin co-receptor activity via exploitation of the large chemical space to develop chemical tools to optimize or manipulate the auxin-related ligand binding.

Nowadays, NAA and 2,4-D are still the most preferred IAA analogues widely used in many biological experiments to induce auxin responses. IAA is known to be chemically unstable (Yamakawa *et al.*, 1979; Dunlap and Robacker, 1988; Nissen and Sutter, 1990) and is photodegraded in culture media and metabolized rapidly *in planta*. NAA and 2,4-D can induce most of the same plant responses as IAA, while being more stable and effective due to much reduced metabolism by the plant (Jackson *et al.*, 2001; Staswick *et al.*, 2005; Peat *et al.*, 2012; Eyer *et al.*, 2016). Moreover, NAA is more lipophilic, easily taken up by diffusion and actively transported by auxin efflux carriers but almost incompatible with influx carriers, making it a suitable tool as an efflux carrier ‘marker’. By contrast, 2,4-D is not lipophilic and its uptake is mostly mediated by the auxin influx carriers, while it is rather poor substrate for auxin efflux carriers, being the influx carrier ‘marker’ (Delbarre *et al.*, 1996; Simon *et al.*, 2013). Although binding affinities for IAA, NAA and 2,4-D for all known auxin co-receptor combinations are unknown, available biochemical data for TIR1–IAA7 and AFB5–IAA7 show that NAA and 2,4-D have similar binding affinities for each of the co-receptors, which are only half of that of the natural auxin IAA (Lee *et al.*, 2014), implying a generally weaker binding for NAA and 2,4-D than for IAA. However, NAA and 2,4-D often appear to be more effective than IAA in terms of induction of developmental responses (Skůpa *et al.*, 2014; Peterson *et al.*, 2016), because both are less metabolized and 2,4-D is also less exported, as discussed above. Another interesting physiological difference between IAA and 2,4-D is that 2,4-D shows the highest activity on promoting cell division in suspension culture-grown tobacco BY-2 cells among the 12 auxinic compounds tested, including IAA, which surprisingly shows no stimulatory effect at all (Simon *et al.*, 2013). This explains why 2,4-D is the common auxin analogue used in the standard culture medium for BY-2 cells.

Characterization of molecular components of the auxin signaling pathway has been advanced by genetic mutation analyses in the model plant species Arabidopsis. Early chemical genetic screens in 1980s chose the highly stable 2,4-D in place of IAA to look for auxin resistance of mutants. Molecular identification of a series of loci named *AUXIN-RESISTANT* (*AXR*) arising from the screens, including *AXR1* to -*3*, *AXR5*, and *AXR6*, revealed the components of the ubiquitin proteasome pathway and the Aux/IAA transcriptional repressors as being involved in auxin response (Maher and Martindale, 1980; Estelle and Somerville, 1987; Leyser *et al.*, 1993; Hellmann *et al.*, 2003; Woodward and Bartel, 2005). Those investigations, together with the identification of TIR1/AFBs as auxin receptors and ARFs as transcriptional factors regulating auxin-responsive genes, eventually revealed that a derepression mechanism controlled by ubiquitin-mediated proteolysis via SCF^TIR1/AFB^ plays a central role in the transcriptional pathway of auxin signaling (Weijers and Wagner, 2016).

As described earlier, TIR1 and AFB1–3 proteins are similar to each other, while AFB4 and AFB5 are distinct from the others due to their amino-terminal extensions (Parry *et al.*, 2009). Ligand specificity of these two types of auxin receptors has been revealed by chemical genetic screens and/or biochemical assays using various synthetic auxin analogues. Like the endogenous auxin IAA, 2,4-D and its structural analogues, such as 2-(2,4-dichlorophenoxy) propionic acid and 2,4,5-trichlorophenoxyacetic acid (2,4,5-T) (Fig. 1), exert an auxin response mainly via TIR1 type receptors (Simon *et al.*, 2013; Lee *et al.*, 2014). To identify upstream components of the auxin response, a chemical genetic screen was designed to isolate mutations exhibiting differential resistance to 2,4-D and picolinate auxins using a potent picolinate auxin DAS534 (Fig. 1), an analogue of the auxinic herbicide picloram (Fig. 1), as the probe. One of the two mutants identified results from the disruption of *AFB5*. Mutations in this gene and its close homologue *AFB4* confer selective resistance to picolinate auxins, especially picloram, while conferring very low or no cross-resistance to 2,4-D or IAA (Walsh *et al.*, 2006; Prigge *et al.*, 2016). These results imply that the picolinate-type auxin analogues are selectively recognized by AFB4/5–Aux/IAA co-receptors. This is further confirmed by biochemical studies showing that picolinate-type compounds, like picloram, fluoxypyr, trichlopyr, and DAS534, bind to AFB5–IAA7 as efficiently as IAA (Calderón-Villalobos *et al.*, 2012; Lee *et al.*, 2014). Intriguingly, quinclorac (Fig. 1), a quinoline-2-carboxylate auxin analogue, also shows high specificity towards the AFB5–Aux/IAA co-receptor. Thus, those auxin analogues, such as 2,4,5-T and picloram or quinclorac, can be used to probe the different auxin receptor proteins. For example, a group of researchers clarified the acid growth theory (Jacobs and Ray, 1976; Rayle and Cleland, 1977) for the classical auxin response of rapid cell elongation by comprehensive analyses (Fendrych *et al.*, 2016). They proved that the rapid cell wall acidification and cell elongation induced by auxin require TIR1/AFB–Aux/IAA signaling, because interruption of the co-receptor activity by the stabilized Aux/IAA with a mutation in the DII domain completely abolishes the effects (Fendrych *et al.*, 2016). To corroborate this conclusion from the angle of TIR1/AFB, they took advantage of the specific interaction between the synthetic auxin picloram and its cognate receptor AFB5, instead of using the high order *tir1*/*afb* mutants that are strongly defective in development and thus difficult for physiological assessment (Dharmasiri *et al.*, 2005*b*; Parry *et al.*, 2009). Picloram induces cell elongation in the hypocotyl in wild-type background, whereas the *afb5-5* mutant shows a clear reduction in this response to picloram, further confirming the involvement of the TIR1/AFB–Aux/IAA auxin co-receptor complex in cell elongation (Fendrych *et al.*, 2016). Here, the use of the auxin analogue picloram as a chemical tool, in combination with the *afb5-5* mutant that has normal morphology but shows decreased response to picloram, provided an elegant way to confirm the crucial role of the TIR1/AFB pathway in auxin-induced hypocotyl elongation (Fendrych *et al.*, 2016). Furthermore, IAA only slightly prefers TIR1 to AFB5, as shown by similar binding affinities (Calderón-Villalobos *et al.*, 2012). By contrast, picloram, a synthetic molecule not naturally present in cells, significantly prefers AFB5 over TIR1 with around 70 times higher affinity (Calderón-Villalobos *et al.*, 2012). From the perspective of evolution, this implies the existence of a selection for more than a single binding pattern on AFB5, suggesting that there might be additional endogenous ligands still to be identified (Lee *et al.*, 2014).

### Proauxins identified by chemical biology

Since the first application of chemical biology to basic auxin research less than two decades ago, great advances have been made not only in the mechanistic understanding of auxin action, but also in the different ways that this powerful approach can be used to interrogate the auxin mystery (De Rybel *et al.*, 2009; Ma and Robert, 2014; Rigal *et al.*, 2014; Doyle *et al.*, 2015). With the current availability of various novel genetic tools, automatic phenotyping and high-throughput technologies, and a collection of synthetic and natural bioactive compound libraries, the wide chemical space is being explored by chemical genomics to develop useful small-molecule tools for auxin research (Hicks and Raikhel, 2012; Dejonghe and Russinova, 2017). Auxin agonists affecting physiological processes of interest in a spatially or temporally controlled fashion would be useful tools to dissect the complexity of auxin signaling. To this end, a collection of auxin agonists have been identified through forward chemical genetic screens. A subset of these chemicals, identified by two independent phenotype-based screens as described below, has been shown to function as ‘proauxins’ akin to prodrugs. The potent compound 602 and compound 533 (Fig. 1), two representatives of 100 positive molecules stimulating hypocotyl elongation in one screen system (Savaldi-Goldstein *et al.*, 2008), and the potent WH7 (Fig. 1), one of 13 positive molecules inhibiting primary root elongation in another screen (Christian *et al.*, 2008), share common structural characteristics: a bipartite structure consisting of a synthetic auxin, herein the well-characterized 2,4-D or its variant, conjugated to a hydrophobic and/or heterocyclic moiety (a chemical masking agent) via an amide linkage (the amide conjugate). On the one hand, the intact molecules are generally more lipophilic than auxin over the physiological pH range of 5.0–7.5, and therefore cell membrane permeant and subject to more efficient cellular uptake. On the other hand, upon arriving at the site of action, the functional auxin is locally released via hydrolysis of the amide bond (Christian *et al.*, 2008; Savaldi-Goldstein *et al.*, 2008). In this way, the compound 602 is able to induce Arabidopsis hypocotyl elongation in light while having no obvious effect on primary root length. It has been shown that compound 602 is efficiently absorbed into the hypocotyls where it is hydrolysed to release 4-chloro-3,5-dimethylphenoxyacetic acid (602-UC) (Fig. 1), a 2,4-D analogue with weak auxin activity. This finding is in agreement with the fact that hypocotyl elongation in light is sensitive to low levels of auxin (Savaldi-Goldstein *et al.*, 2008). Due to such a proauxin’s selective activity in specific plant tissues that are otherwise inaccessible by the application of auxin, proauxins provide a useful new approach to address unanswered questions on specific aspects of auxin signaling. Characterization of mutants with altered response to proauxins via either forward or reverse genetics can be used to discover new molecular components specific to the target signaling cascades controlling specific aspects of the complex auxin responses.

Another hypothesis about proauxin is that the intact molecule, like its cognate auxin moiety, might be perceived by the TIR1/AFB–Aux/IAA auxin co-receptor and generate its own activity. This intact activity, in addition to the locally released auxin activity as described above, might also contribute to the differential response eventually observed. There are several lines of evidence to support this hypothesis. First, although proauxin is hydrolysed *in planta*, the hydrolysis efficiency is much less than 100%, implying the existence of the intact molecule in the target tissues. Second, the partial promiscuity of the auxin-binding site might potentially allow the docking of proauxin, which is the amide conjugate of 2,4-D and therefore fulfils the structural requirements of binding. Third, in the original pull-down assay, the proauxins compound 602 and compound 533 could both enhance the interaction between TIR1-myc and GST-AUX/IAA7, though with different efficiency, implying the ability of these compounds to bind to the auxin co-receptors (Savaldi-Goldstein *et al.*, 2008). These lines of evidence support the hypothesis that the intact molecule of proauxin can generate its own activity by binding to the TIR1/AFB–Aux/IAA auxin co-receptor complex. Nonetheless, a molecule’s binding to the co-receptor complex does not necessarily lead to the increased interaction between TIR1/AFB and Aux/IAA. According to the molecular glue model, auxin has to fill the cavity at the imperfect protein interface to create a continuous hydrophobic protein interaction surface without causing steric hindrance. If a molecule can bind to TIR1/AFB with reasonable affinity but meanwhile causes structural hindrance to impede the interaction with Aux/IAA, it will function as an auxin antagonist. This is in agreement with the recent finding that the size and structure of the side chain of an auxin-related molecule determines whether it is an auxin agonist or antagonist (Hayashi *et al.*, 2008). The same might apply for proauxin. The biochemical properties of various TIR1/AFB–Aux/IAA co-receptors are different and the co-receptors show binding selectivity towards different auxins (Calderón-Villalobos *et al.*, 2012; Lee *et al.*, 2014; Shimizu-Mitao and Kakimoto, 2014). A proauxin might either stabilize or block the interaction between TIR1/AFB and Aux/IAA depending on its structural impact on the interaction. One can envision that, depending on the repertoire of the auxin co-receptor combinations present in the target tissue and the structural impacts of a proauxin (including the released cognate auxin) on them, the application of the proauxin will create a co-receptor assembly pattern within the tissue where a specific response will occur. The signaling cascade incurred by the proauxin can be unraveled by genetic analysis, as described before, and/or genomic approaches such as, but not limited to, transcriptomics. Thus, a connection between the auxin co-receptor assembly pattern, the corresponding signaling cascade, and the resulting response can be established by using the proauxin as a probe. There are several reviews describing general chemical genomic strategies in plants, based on which proauxins could be utilized (Kaschani and van der Hoorn, 2007; Robert *et al.*, 2009; Hicks and Raikhel, 2012; Dejonghe and Russinova, 2017).

### Caged auxins for spatiotemporal control of auxin response

**Fig. 2.**
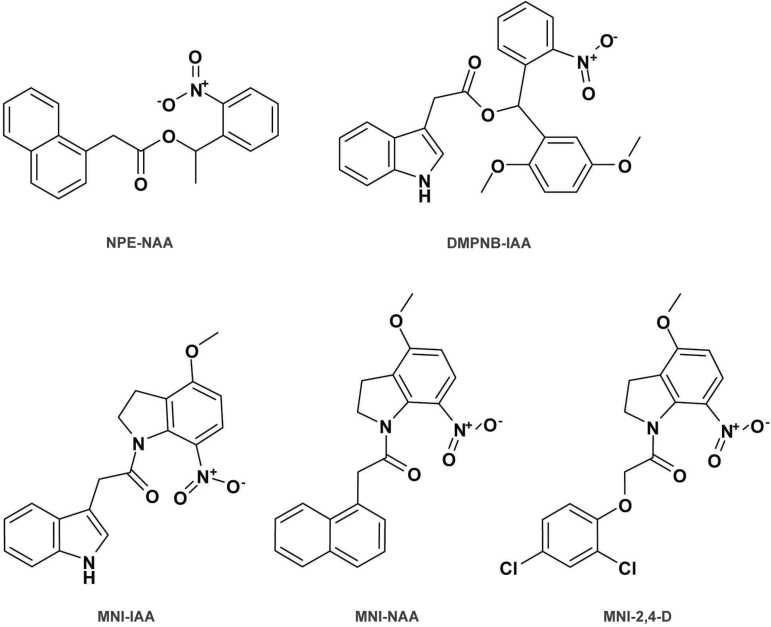
Structures of caged auxins. The overview of caged auxins includes 1-(2-nitrophenyl)ethyl NAA (NPE-NAA), (2,5-dimethoxyphenyl)(2-nitrobenzyl) indole 3-acetate (DMPNB-IAA), and 4-methoxy-7-nitroindolinyl (MNI)-caged IAA, NAA, and 2,4-D (MNI-IAA, MNI-NAA, and MNI-2,4-D).

A key issue for dissecting cell-specific auxin responses is how to control auxin signaling at a cellular level in a spatiotemporal way. The use of caged auxin molecules provides a powerful approach to temporally control auxin levels at a tissue, organ or even cellular level (Kusaka *et al.*, 2009). A caged compound is a trapped bioactive molecule chemically inactivated by coupling with a photo-cleavable protecting group (caging group) and can rapidly release the original active molecule upon photolysis. The intracellular auxin response can be precisely and instantly manipulated by uncaging/releasing the caged auxin via photo-irradiation, because light is easily controllable in terms of period, area, and strength. The optical devices that can be used for irradiation include a flash lamp, hand-held UV light, fluorescence microscope, or confocal microscope with UV laser. The first caged auxin that was produced is 2-nitrophenylethyl (NPE)-caged NAA (Fig. 2), which is limited in usage due to releasing free NAA even without photolysis, because the NPE caging group, originally used for mammalian biology, is easily hydrolysed by plant esterases (Ward and Beale, 1995). Later, an esterase-resistant caging group was used to create 2′,5′-dimethoxyphenyl-2-nitrobenzyl-caged auxins (Fig. 2), which display increased stability (Kusaka *et al.*, 2009). Recently, a group of 4-methoxy-7-nitroindolinyl (MNI)-caged auxins (Fig. 2) has been synthesized and is available for auxin research. They are highly stable both *in vivo* and *in vitro*, because the carboxyl group of the auxin is protected by a secondary amine of the MNI caging group. Moreover, the MNI caging group, which was originally developed for the two-photon uncaging system, provides high spatiotemporal control of photolysis at two-photon cross-section in comparison with one-photon uncaging. MNI-caged auxins have been shown to be able to spatiotemporally control auxin-responsive gene expression and auxin-related physiological responses by controlling light illumination (Hayashi *et al.*, 2015). However, one potential problem for the use of caged auxins is that to achieve spatiotemporal control of photolysis, the plant samples have to be kept in the dark after illumination to avoid unwanted uncaging. Additionally, responses to light have to be taken into consideration when the light effect on an assay has to be accounted for.

### Classical auxin antagonists

**Fig. 3.**
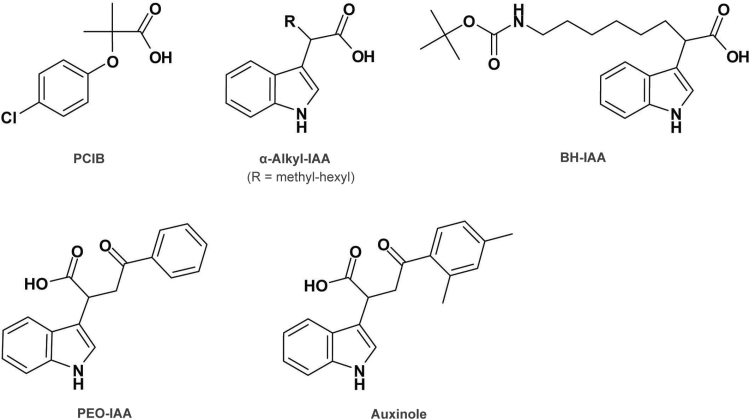
Structural formulae of auxin antagonists. The chemical names for the displayed compounds are: *p*-chlorophenoxyisobutyric acid (PCIB), *tert*-butoxycarbonylaminohexyl-IAA (BH-IAA), α-(phenylethyl-2-oxo)-IAA (PEO-IAA), and α-(2,4-dimethylphenylethyl-2-oxo)-IAA (auxinole).

Besides the aforementioned auxin analogues or agonists, auxin antagonists are also effective tools to study auxin response. By a modern definition, auxin antagonists refer to molecules that can dock in the auxin binding pocket with a reasonable affinity but either do not enhance or even hinder the Aux/IAA interaction (De Rybel *et al.*, 2009). Among the classical auxin antagonists, *p*-chlorophenoxyisobutyric acid (PCIB) (Fig. 3) has been widely used to inhibit auxin signaling and is better characterized at the molecular level (Oono *et al.*, 2003). PCIB was originally identified as an auxin analogue that has a much reduced auxin activity but it can be used to antagonize auxin action by competing at the auxin binding site (Burström, 1950; McRae *et al.*, 1953). Later, PCIB was shown to inhibit auxin (IAA, NAA, and 2,4-D)-induced gene expression that is mediated by the SCF^TIR1/AFB^ pathway, through stabilizing Aux/IAA proteins (Oono *et al.*, 2003). To identify novel factors involved upstream of Aux/IAA degradation in the auxin signaling pathway, a genetic mutation analysis was designed to screen for mutants with altered responses to PCIB (Rahman *et al.*, 2006; Biswas *et al.*, 2007). Among the six identified mutants named *antiauxin-resistant* (*aar*), two are alleles of *tir1* and *cullin1*, key components of the SCF^TIR1/AFB^ complex. *AAR1* encodes SMALL ACIDIC PROTEIN 1 (SMAP1) working downstream of auxin perception but upstream of ubiquitin-dependent Aux/IAA protein degradation (Rahman *et al.*, 2006). *AAR3* encodes a DEFECTIVE IN CULLIN NEDDYLATION-1 (DCN-1)- like protein that might be involved in the regulation of SCF^TIR1/AFB^ activity (Biswas *et al.*, 2007). These studies show that a mutant screening approach with an auxin antagonist has the potential to discover new factors involved in auxin-related signaling pathways that otherwise would not be isolated by using classical auxins.

### Specific auxin antagonists developed by rational structure-based molecular design

The crystal structures of TIR1 in complex with various auxins and an Aux/IAA degron reveal the long-sought mechanism of auxin perception. As described earlier, auxin binds to the base of a pocket within the substrate-interacting region between the TIR1/AFB F-box protein and Aux/IAA, and stabilizes the TIR1/AFB–auxin–Aux/IAA co-receptor complex without inducing any conformational change in the proteins (Tan *et al.*, 2007). This perception model provides a valuable platform for designing and developing auxin agonists and antagonists via a chemical biological approach. To develop specific auxin signaling probes targeting the TIR1/AFB–Aux/IAA co-receptor complex, a series of α-alkyl chains with varying length were introduced to the α-position of IAA (Fig. 3) (Hayashi *et al.*, 2008). Intriguingly, it was found that the length of the side chain determines the auxin activity. For example, IAA molecules with short chains, like methyl to propyl chain substitutions, simulate TIR1–Aux/IAA interaction, leading to enhanced Aux/IAA degradation and auxin-related phenotypes. In contrast, while butyl- or longer chain-substituted IAA molecules still bind to TIR1, they significantly prevent TIR1–Aux/IAA interaction, meaning that these compounds function as auxin antagonists. The crystal structures of TIR1 complexed with three α-alkyl-IAAs show clearly that the IAA moieties of the three molecules sit in the auxin-binding pocket of TIR1 in the same way as IAA itself, whereas the longer alkyl chains protrude into the Aux/IAA-binding cavity, blocking the access of Aux/IAA (Hayashi *et al.*, 2008). This mechanism of antagonism supports and expands the molecular glue model.

Among the first generation of auxin antagonists, *tert*-butoxycarbonylaminohexyl-IAA (BH-IAA) (Fig. 3) is a potent molecule that has been used to confirm that the SCF^TIR1^–Aux/IAA pathway is conserved between lower and higher land plants (Hayashi *et al.*, 2008). To further improve the binding affinity of auxin antagonists for TIR1 to a much higher level than that of the endogenous IAA, an approach was adopted using rational design based on the crystal structure of the TIR1–BH-IAA complex and *in silico* virtual screening of TIR1 ligands from chemical libraries. The initial virtual screening identified α-(phenylethyl-2-oxo)-IAA (PEO-IAA) (Fig. 3) as a very potent candidate compound. The methylation of the phenyl ring of PEO-IAA greatly increased the auxin binding inhibitory activity, leading to the synthesis of the final optimized compound auxinole (α-(2,4-dimethylphenylethyl-2-oxo)-IAA) (Fig. 3) (Hayashi *et al.*, 2012). Molecular docking analysis of auxinole and PEO-IAA predicts that the phenyl ring of both ligands effectively prevents the access of the DII motif in the Aux/IAA protein. Consistent with this predicted mode of action, auxinole and PEO-IAA have been shown to competitively inhibit various auxin responses *in planta*, by blocking the formation of the TIR1–IAA–Aux/IAA complex and stabilizing Aux/IAA repressors (Hayashi *et al.*, 2012). Nowadays, auxinole and PEO-IAA are widely used to reversibly block auxin action, providing novel and powerful tools for chemical biological analysis of auxin-regulated processes in a wide range of plant species (Tatsuki *et al.*, 2013; Procko *et al.*, 2014; Fendrych *et al.*, 2016).

## Conclusions

The continuous advent of technically advanced scientific tools has raised the quality of basic plant research to an unprecedented level. Undoubtedly, the diverse array of bioactive small molecules developed by chemical biology, targeting almost every aspect of auxin action, have enriched the tool box for auxin biology. The simple molecule of auxin regulates a large number of developmental processes via reprogramming gene transcription through the short SCF^TIR1/AFB^–Aux/IAA–ARF nuclear signaling pathway (Weijers and Wagner, 2016). This is, to a large extent, attributable to the diversity of the pathway conferred by the intricate combinatorial protein interactions among the members of the three central actors, contributing to the specificity of auxin response (Wang and Estelle, 2014). In particular, biochemical and biophysical studies of key auxin signaling proteins have shown that the Aux/IAA proteins are central and dynamic regulators of auxin signaling and their degradation is crucial for auxin action (Havens *et al.*, 2012; Pierre-Jerome *et al.*, 2014). The rate of Aux/IAA degradation is dependent on the TIR1/AFB–Aux/IAA co-receptor complexes (Havens *et al.*, 2012), which exhibit different affinities for different auxin-related compounds (Lee *et al.*, 2014; Shimizu-Mitao and Kakimoto, 2014). Due to the inherent complexity of the auxin co-receptors, discrimination of their specific roles in auxin signaling requires more elegant and effective tools than can be offered by genetics and genomics alone. The increased use of bioactive small molecules as chemical tools targeting the TIR1/AFB–Aux/IAA co-receptor complexes has produced novel insights into the molecular basis of transcriptional auxin signaling. The crystallographic data of the TIR1/AFB–auxin–Aux/IAA ternary complex allow novel selective auxin agonists and antagonists to be rationally designed based on protein structure. Those compounds will serve as valuable tools for dissecting the complexity and diversity of auxin response over a broad range of species, disclosing new players involved in the fine-tuning of the core nuclear signaling module, and deciphering the auxin crosstalk with other hormones or environmental signals. Ultimately, these data will provide us with a comprehensive insight into how auxin integrates endogenous and environmental clues to regulate plant growth and development, leading to new and better ways to engineer plant development for advance applications.

## Abbreviations:

2,4,5-T: 2,4,5-trichlorophenoxyacetic acid
2,4-D: 2,4-dichlorophenoxyacetic acid
4-Cl-IAA: 4-chloroindole-3-acetic acid
AAR: ANTIAUXIN-RESISTANT
ABA: abscisic acid
ARF: AUXIN RESPONSE FACTOR
ASK1: ARABIDOPSIS SKP1 HOMOLOGUE
Aux/IAA: AUXIN/INDOLE-3-ACETIC ACID
AXR: AUXIN-RESISTANT
BH-IAA: *tert*-butoxycarbonylaminohexyl-IAA
DAO1: DIOXYGENASE FOR AUXIN OXIDATION 1
GH3: , GRETCHEN HAGEN 3
IAA: indole-3-acetic acid
IBA: indole-3-butyric acid
LRR: leucine rich repeats
MNI: 4-methoxy-7-nitroindolinyl
NAA: naphthalene-1-acetic acid
PAA: phenylacetic acid
PCIB: *p*-chlorophenoxyisobutyric acid
PEO-IAA: α-(phenylethyl-2-oxo)-IAA
SCF: SKP1/CULLIN1/F-Box
SKP1: SUPPRESSOR OF KINETOCHORE PROTEIN 1
TIR1/AFB: TRANSPORT INHIBITOR RESISTANT1/AUXIN SIGNALING F-BOX.
